# Acute limb ischaemia revealing AL cardiac amyloidosis despite sustained sinus rhythm: a case report

**DOI:** 10.1093/ehjcr/ytag662

**Published:** 2026-09-02

**Authors:** Shuhei Osumi, Daisuke Tomioka, Naofumi Oyamada, Toshikazu Jinnai, Kazuaki Kaitani

**Affiliations:** Department of Cardiology, Otsu Red Cross Hospital, 1-1-35, Nagara-cho, Otsu-city, Shiga 525-8511, Japan; Department of Cardiology, Otsu Red Cross Hospital, 1-1-35, Nagara-cho, Otsu-city, Shiga 525-8511, Japan; Department of Cardiovascular Medicine, Shiga University of Medical Science, Tsukinowa, Setatsukinowa-Cho, Otsu-city, Shiga 520-2192, Japan; Department of Cardiology, Otsu Red Cross Hospital, 1-1-35, Nagara-cho, Otsu-city, Shiga 525-8511, Japan; Department of Cardiology, Otsu Red Cross Hospital, 1-1-35, Nagara-cho, Otsu-city, Shiga 525-8511, Japan; Department of Cardiology, Otsu Red Cross Hospital, 1-1-35, Nagara-cho, Otsu-city, Shiga 525-8511, Japan

**Keywords:** Case report, AL amyloidosis, Cardiac amyloidosis, Acute limb ischaemia, Thromboembolism, Sinus rhythm, Atrial mechanical dysfunction

## Abstract

**Background:**

Thromboembolic complications are well recognized in cardiac amyloidosis and are traditionally attributed to atrial fibrillation (AF). However, patients with immunoglobulin light-chain (AL) cardiac amyloidosis remain at substantial thromboembolic risk, even in sinus rhythm, presumably due to atrial mechanical dysfunction. Acute limb ischaemia as the first clinical manifestation of AL cardiac amyloidosis is exceedingly rare.

**Case summary:**

A 64-year-old man presented with sudden-onset pain and coldness in the left lower limb. Computed tomography revealed acute occlusion of the left popliteal artery, and an emergency surgical thrombectomy was successfully performed. Electrocardiography showed sinus rhythm, and continuous monitoring during hospitalization detected no atrial fibrillation. Transthoracic echocardiography demonstrated concentric ventricular wall thickening and reduced transmitral A-wave velocity despite preserved sinus rhythm and generally normal left atrial size, suggesting impaired atrial mechanical function. Cardiac magnetic resonance imaging revealed diffuse subendocardial late gadolinium enhancement extending into the left atrial myocardium. Right ventricular endomyocardial biopsy confirmed AL amyloidosis. The patient was accordingly diagnosed with AL cardiac amyloidosis, complicated by systemic arterial thromboembolism. Daratumumab-based therapy and anticoagulation were initiated, resulting in no recurrence of embolic events and excellent functional recovery during follow-up.

**Discussion:**

This case highlights that AL cardiac amyloidosis can cause clinically significant systemic arterial thromboembolism, even in apparent sinus rhythm. Atrial mechanical dysfunction due to amyloid infiltration, rather than atrial fibrillation, appears to be a key mechanism. Clinicians should consider cardiac amyloidosis in patients with embolic events of unknown origin, particularly when imaging findings suggest infiltrative cardiomyopathy.

Learning points

- AL cardiac amyloidosis can cause systemic arterial thromboembolism likely because of atrial mechanical dysfunction from amyloid infiltration.
- Doppler indices and multimodality imaging may reveal impaired atrial contractility despite preserved sinus rhythm and normal left atrial size.
- Cardiac amyloidosis should be considered in patients with embolic events of unknown origin when imaging findings suggest infiltrative cardiomyopathy.

## Introduction

Systemic immunoglobulin light-chain (AL) amyloidosis is a rare multisystem disorder caused by the extracellular deposition of misfolded monoclonal light-chain proteins.^1^ Cardiac involvement occurs in approximately half of the patients and is the principal prognostic determinant.^1,2^ In addition to progressive heart failure, thromboembolic complications are a major cause of morbidity and mortality in AL cardiac amyloidosis.^3,4^

Traditionally, systemic arterial embolism has been attributed to atrial fibrillation (AF). However, accumulating evidence indicates that patients with cardiac amyloidosis are also at an increased risk of intracardiac thrombosis and systemic embolism, even while maintaining sinus rhythm.^3,5,6^ This paradox has been attributed to atrial mechanical dysfunction related to amyloid infiltration.^5,7,8^ However, acute limb ischaemia as the first clinical manifestation of AL cardiac amyloidosis remains exceedingly rare.^9,10^

## Summary figure

**Figure:**
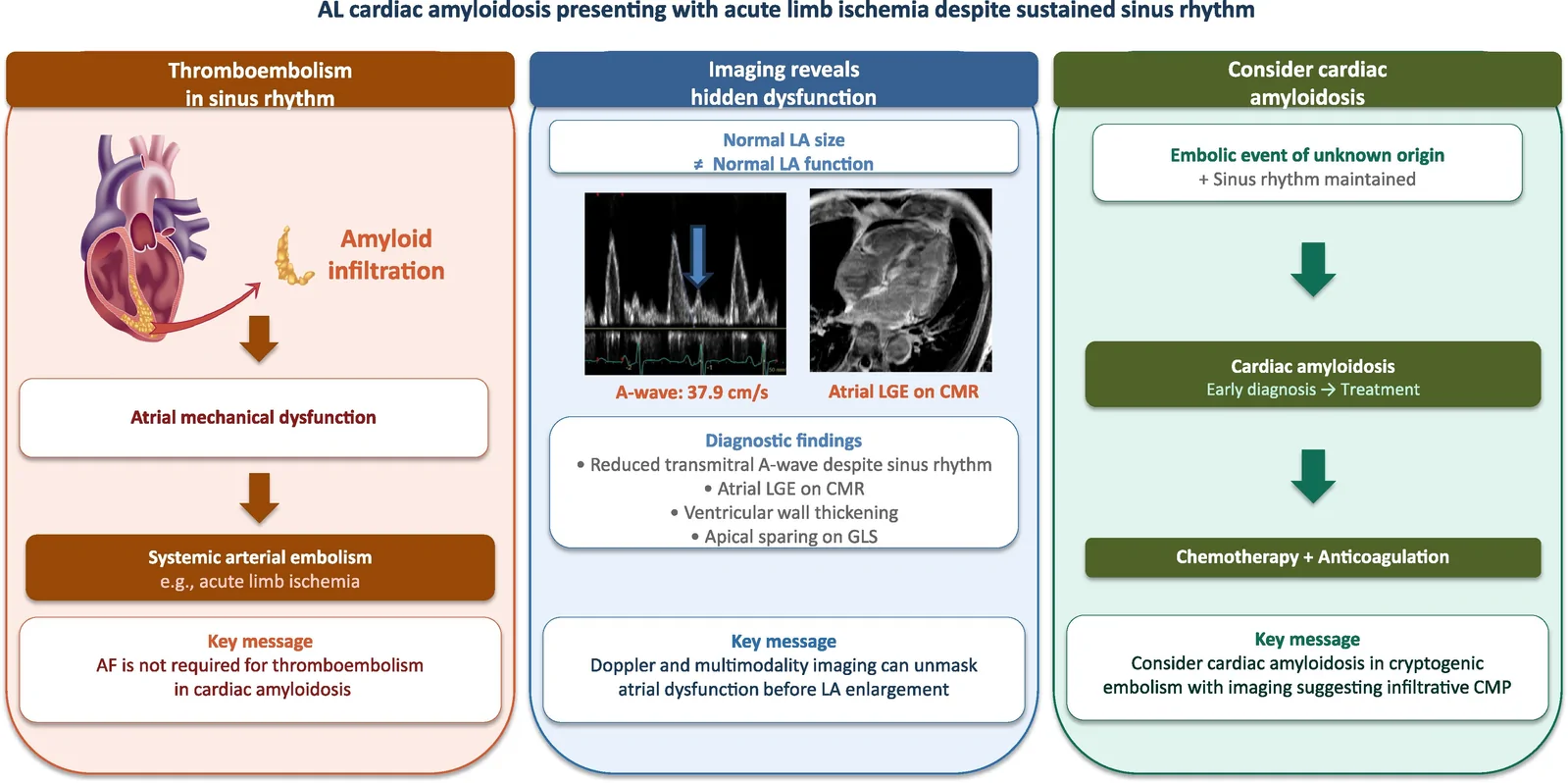


## Case presentation

A 64-year-old man with no known history of cardiovascular disease, AF, thromboembolic events, or malignancy presented to the emergency department with sudden-onset pain, numbness, and coldness of the left lower limb. He was a non-smoker and had no history of hypertension, diabetes mellitus, or dyslipidaemia. He had no family history of cardiomyopathy or amyloidosis and was not taking any regular medication. On arrival, physical examination revealed a cold and pale left lower extremity with absent popliteal and distal pulses; the contralateral limb was unremarkable. Vital signs were stable (blood pressure, 120/70 mmHg; heart rate, ∼70 beats/min; and oxygen saturation, >95% on room air). Cardiac auscultation revealed no significant murmurs, and the lung fields were clear. No peripheral oedema, macroglossia, purpura, or carpal tunnel signs were observed.

**Figure 1:**
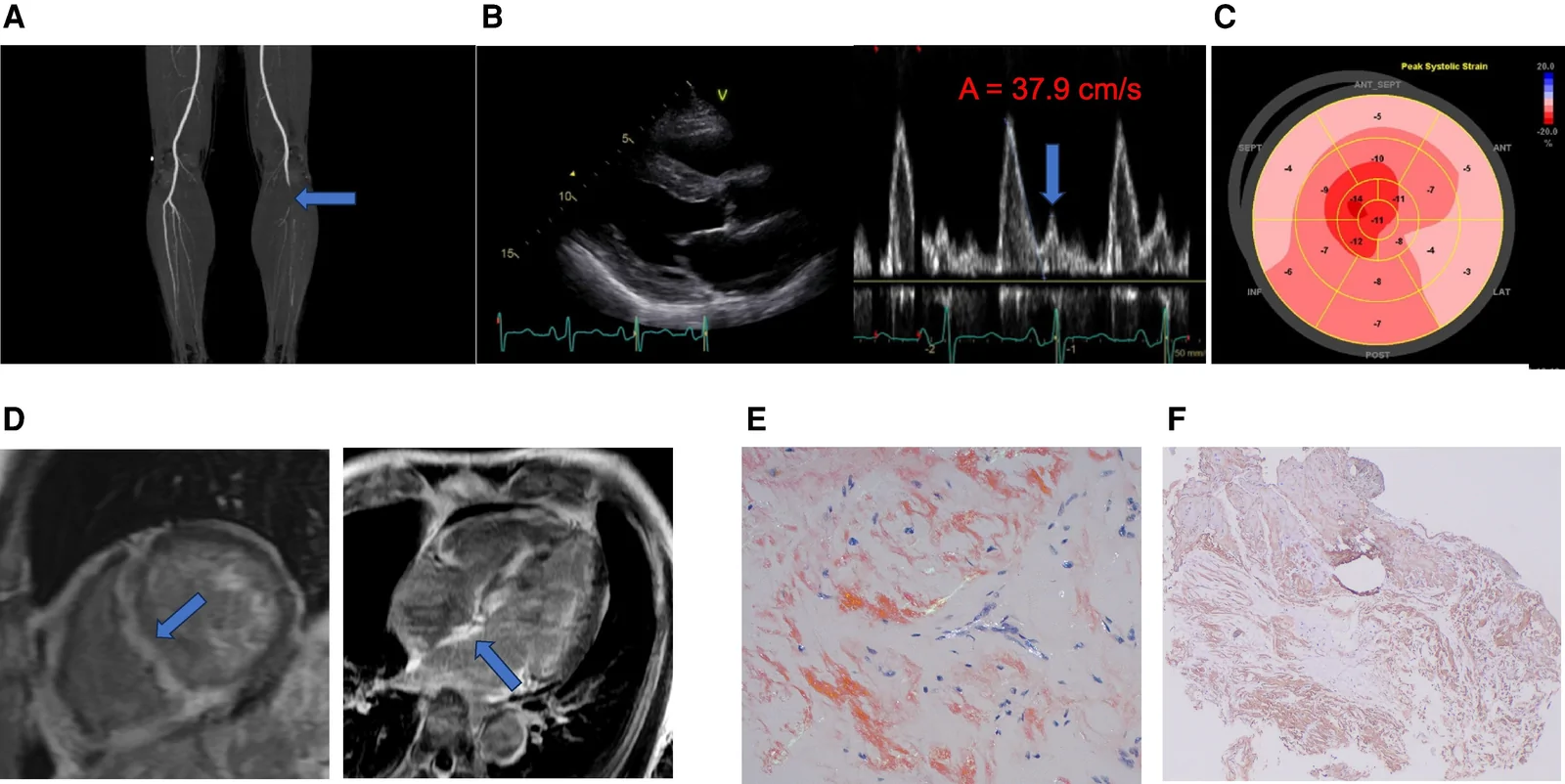
Multimodality diagnostic findings in our patient with AL cardiac amyloidosis presenting with acute limb ischaemia. (*A*) Contrast-enhanced computed tomography of the lower extremities demonstrating acute occlusion of the left popliteal artery (arrow) without underlying atherosclerotic disease. (*B*) Transthoracic echocardiography showing concentric ventricular wall thickening with increased myocardial echogenicity (left panel). Transmitral Doppler demonstrating reduced A-wave velocity (37.9 cm/s; arrow) despite sinus rhythm, indicating impaired atrial mechanical function (middle panel). (*C*) Global longitudinal strain bull’s eye map demonstrating relative apical sparing, a characteristic pattern of cardiac amyloidosis. (*D*) Cardiac magnetic resonance imaging: short-axis view (left) and four-chamber view (right) showing diffuse subendocardial late gadolinium enhancement involving both ventricles and extending into the atrial myocardium (arrows). (*E*) Endomyocardial biopsy specimen showing Congo red–positive amyloid deposits with characteristic orange-red staining. (*F*) Immunohistochemical staining confirming lambda light-chain amyloid deposition.

Transthoracic echocardiography revealed concentric thickening of both ventricular walls, with an interventricular septal thickness of 14.4 mm and diffusely increased myocardial echogenicity. The left ventricular ejection fraction was preserved at 56%. Global longitudinal strain demonstrated relative apical sparing, a characteristic finding in cardiac amyloidosis (*Figure 1B, C*). Despite a maintained sinus rhythm, transmitral Doppler echocardiography demonstrated an E-wave velocity of 91.7 cm/s and a markedly reduced A-wave velocity of 37.9 cm/s (normal range approximately 40–90 cm/s depending on age), with an E/A ratio of 2.4 and an E/e’ ratio of 27, consistent with grade III (restrictive) diastolic dysfunction, indicating impaired atrial contractile function. The left atrial size was nearly normal despite evidence of atrial mechanical dysfunction.

Given the unexplained systemic embolism in sinus rhythm and echocardiographic features suggestive of infiltrative cardiomyopathy, further diagnostic evaluation was performed. 99mTc-pyrophosphate scintigraphy showed no significant myocardial uptake, making transthyretin amyloidosis unlikely. Cardiac magnetic resonance imaging (CMR) revealed difficulty in achieving appropriate myocardial nulling and diffuse subendocardial to transmural late gadolinium enhancement involving both ventricles, extending into the atrial myocardium, which was highly suggestive of cardiac amyloidosis (*Figure 1D*). No intracardiac thrombus was identified on CMR. Native T1 mapping and extracellular volume quantification were not available at our institution during the evaluation period.

A right ventricular endomyocardial biopsy was performed rather than abdominal fat pad aspiration because confirmation of direct cardiac amyloid involvement was considered essential for determining the indication for systemic chemotherapy. Histopathological examination revealed Congo red–positive amyloid deposits with apple-green birefringence under polarized light. Immunohistochemical analysis identified immunoglobulin light-chain amyloid, confirming the diagnosis of AL amyloidosis with cardiac involvement (*Figure 1E, F*). Serum free light-chain analysis revealed markedly elevated lambda light chains (κ, 6.8 mg/L [normal: 3.3–19.4 mg/L]; λ, 1010 mg/L [normal: 5.7–26.3 mg/L]; κ/λ ratio, 0.01), with a difference between involved and uninvolved free light chains of 1003.2 mg/L. Bone marrow biopsy demonstrated 10% clonal plasma cells. No lytic bone lesions, hypercalcaemia, renal impairment, or anaemia attributable to a plasma cell neoplasm were identified; therefore, the diagnosis was primary (isolated) AL amyloidosis rather than multiple myeloma. The patient was accordingly diagnosed with AL cardiac amyloidosis complicated by systemic arterial thromboembolism. The patient was classified with stage III disease following the revised Mayo 2012 staging system.

Chemotherapy with daratumumab, bortezomib, cyclophosphamide, and dexamethasone (Dara-VCD) was initiated 1 month later in collaboration with the haematology team. Anticoagulation with apixaban was selected after multidisciplinary discussion. Apixaban was initiated for the secondary prevention of thromboembolism. Holter electrocardiography performed during hospitalization confirmed sinus rhythm without paroxysmal atrial fibrillation or sustained atrial tachyarrhythmia. The patient remained in sinus rhythm during follow-up, as assessed by 12-lead electrocardiography at each outpatient visit, with no recurrence of thromboembolic events or major bleeding. His functional capacity improved and was maintained at an Eastern Cooperative Oncology Group performance status score of 0 at the 6-month follow-up.

## Discussion

In the present case, transmitral Doppler echocardiography demonstrated a restrictive filling pattern (E/A ratio 2.4, E/e’ 27) with a disproportionately reduced transmitral A-wave velocity despite maintained sinus rhythm, suggesting impaired atrial contractile function^11^ beyond what would be expected from elevated filling pressures alone. CMR revealed atrial late gadolinium enhancement consistent with direct atrial involvement, a finding previously associated with structural myocardial infiltration.^12^ Multimodality imaging has also revealed rare thrombotic manifestations such as coronary sinus thrombosis.^13^ These findings support that atrial functional failure, rather than electrical rhythm disturbance alone, may be a clinically relevant substrate for systemic embolism in AL cardiac amyloidosis.

The optimal anticoagulation strategy for cardiac amyloidosis remains an unresolved clinical question, with no randomized data comparing anticoagulant agents or examining the optimal duration of therapy in this population.^7,14^ Current expert consensus statements suggest that anticoagulation should be considered in patients with documented intracardiac thrombus or systemic embolism regardless of rhythm status. In our patient, a direct oral anticoagulant was selected after multidisciplinary discussion, primarily to avoid pharmacokinetic interactions between warfarin and the Dara-VCD chemotherapy regimen, particularly dexamethasone-induced CYP3A4 induction and resultant INR variability. Whether anticoagulation can be safely discontinued after sustained haematologic remission with the recovery of atrial mechanical function remains unknown and warrants investigation in future prospective studies. This report does not advocate a specific regimen but underscores the need for individualized decision-making in this high-risk population.

From a clinical perspective, this case highlights that systemic embolism in sinus rhythm should not automatically exclude cardiac sources of embolism, particularly in patients with unexplained ventricular wall thickening or imaging findings suggestive of infiltrative cardiomyopathy. Assessment of atrial function using Doppler parameters and multimodality imaging may provide clinically relevant information beyond rhythm status alone.

Several limitations should be acknowledged. Transoesophageal echocardiography was not performed because patient consent could not be obtained; therefore, left atrial appendage function and intracardiac thrombus could not be definitively assessed.^15^ Although Holter electrocardiography and continuous electrocardiographic monitoring during hospitalization did not detect atrial fibrillation, prolonged ambulatory rhythm monitoring after discharge was not performed. Native T1 mapping and extracellular volume quantification were not available. Although a systemic prothrombotic state associated with plasma cell dyscrasia could have contributed, the imaging findings of atrial mechanical dysfunction and atrial LGE support a cardioembolic mechanism. Nevertheless, the causal relationship between atrial mechanical dysfunction and embolic events cannot be definitively established in a single case report. Future prospective studies with systematic atrial functional assessment, including left atrial strain analysis, are needed to better characterize thromboembolic risk in cardiac amyloidosis patients maintaining sinus rhythm.

In conclusion, AL cardiac amyloidosis can present with systemic arterial thromboembolism even in patients maintaining sinus rhythm. Atrial mechanical dysfunction related to amyloid infiltration may represent an important substrate for thrombogenesis. Clinicians should therefore consider cardiac amyloidosis in patients presenting with embolic events of unknown origin, particularly when imaging findings suggest infiltrative cardiomyopathy.

## Lead author biography


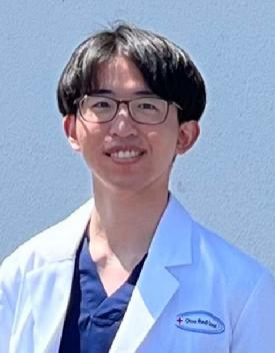
Shuhei Osumi is a physician of Cardiovascular Medicine at Otsu Red Cross Hospital. He achieved a Bachelor’s Degree in Medicine from Kyoto University and works in the general field of cardiovascular medicine.

## Data Availability

The data underlying this article will be shared on reasonable request to the corresponding author.
